# UNDERSTANDING SOCIAL AND CULTURAL DIVERSITIES AND AGING FOR HEALTH AND WELL-BEING IN KOREAN AND KOREAN AMERICANS

**DOI:** 10.1093/geroni/igac059.765

**Published:** 2022-12-20

**Authors:** Seunghye Hong, Michin Hong, Kathryn Braun

## Abstract

Guided by the socio-ecological model and the cultural diversity perspective, this symposium aims to enhance the understanding of critical issues in health and well-being among Koreans and Korean Americans with three primary focuses: aging, social-ecological and multilevel factors, and identifying social and cultural contexts. Five studies examined multilevel factors—individual, relational/interpersonal, community, and societal—that are associated with health and well-being, conducted in Korea as well as in the United States. Study 1 examined psychological well-being among older Koreans, specifically its association with intergenerational relationships and social support using longitudinal multilevel modeling to estimate depression trajectories. Study 2 examined childhood experiences and midlife cultural engagement associations among middle-aged Korean couples, considering the influences of their spouses’ experiences and cultural resources. Study 3 explored the experiences of the nature-based virtual reality program among older Korean Americans, using in-depth interviews and providing an innovative approach using technology as a therapeutic tool. Study 4 examined social determinants of health associated with Korean American immigrants’ willingness for end-of-life discussions and the factors affecting willingness (awareness of hospice, communication with family/doctors, and social isolation). Study 5 examined health insurance coverage and its association with immigration-related factors (English proficiency, generational status, and age at immigration) among Korean Americans using national data. The various health, mental health, and well-being issues in Koreans and Korean Americans will be discussed from contextually responsive approaches. This symposium will provide implications for practices, education, research, and policy to promote health, mental health, and well-being in the Korean and Korean American populations.

